# 3D whole-brain vessel wall cardiovascular magnetic resonance imaging: a study on the reliability in the quantification of intracranial vessel dimensions

**DOI:** 10.1186/s12968-018-0453-z

**Published:** 2018-06-14

**Authors:** Na Zhang, Fan Zhang, Zixin Deng, Qi Yang, Marcio A. Diniz, Shlee S. Song, Konrad H. Schlick, M. Marcel Maya, Nestor Gonzalez, Debiao Li, Hairong Zheng, Xin Liu, Zhaoyang Fan

**Affiliations:** 1 Paul C. Lauterbur Research Center for Biomedical Imaging, Shenzhen Institutes of Advanced Technology, Chinese Academy of Sciences, 1068 Xueyuan Ave., Shenzhen University Town, Shenzhen, 518055 China; 2 Biomedical Imaging Research Institute, Department of Biomedical Sciences, Cedars-Sinai Medical Center, 8700 Beverly Blvd., PACT 400, Los Angeles, CA 90048 USA; 3 Shenzhen College of Advanced Technology, University of Chinese Academy of Sciences, Shenzhen, China; 4 Department of Bioengineering, University of California, Los Angeles, CA USA; 5 Biostatistics and Bioinformatics Research Center, Cedars-Sinai Medical Center, Los Angeles, CA USA; 6 Department of Neurology, Cedars-Sinai Medical Center, Los Angeles, CA USA; 7 Department of Radiology, Cedars-Sinai Medical Center, Los Angeles, CA USA; 8 Department of Neurosurgery, Cedars-Sinai Medical Center, Los Angeles, CA USA; 9 Department of Medicine, University of California, Los Angeles, CA USA

**Keywords:** Intracranial vessel wall morphology, Vessel wall imaging, Whole-brain, Reliability, Magnetic resonance imaging, Intracranial atherosclerotic disease

## Abstract

**Background:**

One of the potentially important applications of three-dimensional (3D) intracranial vessel wall (IVW) cardiovascular magnetic resonance (CMR) is to monitor disease progression and regression via quantitative measurement of IVW morphology during medical management or drug development. However, a prerequisite for this application is to validate that IVW morphologic measurements based on the modality are reliable. In this study we performed comprehensive reliability analysis for the recently proposed whole-brain IVW CMR technique.

**Methods:**

Thirty-four healthy subjects and 10 patients with known intracranial atherosclerotic disease underwent repeat whole-brain IVW CMR scans. In 19 of the 34 subjects, two-dimensional (2D) turbo spin-echo (TSE) scan was performed to serve as a reference for the assessment of vessel dimensions. Lumen and wall volume, normalized wall index, mean and maximum wall thickness were measured in both 3D and 2D IVW CMR images. Scan-rescan, intra-observer, and inter-observer reproducibility of 3D IVW CMR in the quantification of IVW or plaque dimensions were respectively assessed in volunteers and patients as well as for different healthy subjectsub-groups (i.e. < 50 and ≥ 50 years). The agreement in vessel wall and lumen measurements between the 3D technique and the 2D TSE method was also investigated. In addition, the sample size required for future longitudinal clinical studies was calculated.

**Results:**

The intra-class correlation coefficient (ICC) and Bland-Altman plots indicated excellent reproducibility and inter-method agreement for all morphologic measurements (All ICCs > 0.75). In addition, all ICCs of patients were equal to or higher than that of healthy subjects except maximum wall thickness. In volunteers, all ICCs of the age group of ≥50 years were equal to or higher than that of the age group of < 50 years. Normalized wall index and mean and maximum wall thickness were significantly larger in the age group of ≥50 years. To detect 5% - 20% difference between placebo and treatment groups, normalized wall index requires the smallest sample size while lumen volume requires the highest sample size.

**Conclusions:**

Whole-brain 3D IVW CMR is a reliable imaging method for the quantification of intracranial vessel dimensions and could potentially be useful for monitoring plaque progression and regression.

**Electronic supplementary material:**

The online version of this article (10.1186/s12968-018-0453-z) contains supplementary material, which is available to authorized users.

## Background

Intracranial atherosclerotic disease (ICAD) is one of the major causes for cerebrovascular events such as stroke and transient ischemic attack [1, 2]. Luminography imaging, routinely used in the diagnostic workup of ICAD, is restricted to the detection of luminal stenosis, which is, however, not a specific marker for confirming and risk-stratifying atherosclerotic plaques [3]. In contrast, high-resolution black-blood cardiovascular magnetic resonance (CMR) can directly visualize the intracranial vessel wall (IVW) and has demonstrated the potential to characterize plaque features that are intimately associated with clinical events [4–8].

Three-dimensional (3D) turbo spin-echo (TSE) with variable refocusing flip angles, as a black-blood CMR technique, has recently gained growing interest among the IVW imaging research community [9–15]. Compared with a conventionally used two-dimensional (2D) TSE method, the 3D approach provides larger spatial coverage, higher spatial resolution and signal-to-noise ratio (SNR), and the flexibility in image visualization, which are all desirable for visualizing small, tortuous, and deep-seated intracranial arteries. Continued technical improvements are being introduced to the technique, primarily in signal suppression of the cerebrospinal fluid (CSF) [16–19] and arterial blood [17], spatial coverage [16, 18], and scan efficiency [20]. Notably, a whole-brain IVW CMR imaging method was recently developed by incorporating non-selective excitation and a trailing magnetization flip-down module with a commercially available 3D TSE sequence - Sampling Perfection with Application-optimized Contrast using different flip angle Evolutions (SPACE) [18]. Remarkable CSF signal attenuation and enhanced image SNR and T1 contrast weighting make the technique well suited for evaluating vessel wall morphology and revealing plaque features with a characteristic hyper-intense appearance such as intra-plaque hemorrhage and post-contrast wall enhancement. With additional optimization at 3 Tesla, a 3D scan with a whole-brain spatial coverage and isotropic 0.5-mm spatial resolution can be completed within 7–8 min [20]. Such improved imaging efficiency further strengthens its applicability for clinical settings.

One of the potentially important applications of 3D IVW CMR is to monitor ICAD progression and regression via quantitative measurement of vessel wall morphology during medical management or drug development. Demonstrated in extracranial vascular beds, several plaque morphologic measures derived by high-resolution black-blood CMR, such as mean wall thickness, plaque burden, and wall remodeling ratio, may serve as imaging surrogates for therapeutic responses [21–24]. A key prerequisite for 3D IVW CMR to become an imaging tool for longitudinal ICAD assessment is the reliability of the technique in vessel wall and lumen dimension quantification. However, there is a paucity of data reported on the aspect [9, 25].

The purpose of this study was to perform comprehensive reliability analysis for 3D IVW CMR, particularly the recently proposed whole-brain IVW CMR imaging technique [20]. Scan-rescan, intra-observer, and inter-observer reproducibility in the quantification of intracranial vessel dimensions were respectively assessed for healthy subjects and patients with ICAD as well as for different sub-groups (i.e. age < 50 and ≥ 50 years). The agreement in vessel wall and lumen measurements between the 3D technique and the conventionally used 2D TSE method was also investigated in a subgroup of the subjects. In addition, the sample size required for future longitudinal clinical studies was calculated. The findings from this study are expected to indicate the performance of the method in general populations and to provide insights into planning future studies on clinical patients.

## Methods

### Study population

The prospective study was approved by the local institutional review board. Thirty-four healthy subjects (24 males; 14 aged 31–49 years and 20 aged 50–66 years) without known cerebrovascular diseases and 10 patients (7 males; 42–69 years, mean 51.2 years) with known ICAD were recruited. Written informed consent was obtained from all subjects.

### Imaging protocol

All CMR examinations were performed on a 3-Tesla whole-body system (MAGNETOM Verio, Siemens Healthineers, Erlangen, Germany) with a 32-channel head coil. Subjects were scanned in a supine position with a foam padding to minimize head movement. Two repeated 3D IVW CMR scans were performed with an off-table break for healthy subjects and 7 to 11-day intervals for patients [18, 20]. When any of the two scans exhibited motion-related image blurring at the discretion of the CMR technologist, reacquisition was attempted only once to simulate real clinical settings. Relevant imaging parameters were as follows: sagittal imaging orientation, repetition time (TR) /echo time (TE) = 900/15 ms, receiver bandwidth = 488 Hz/pixel, field of view = 170 × 170 × (110–127) mm^3^, matrix size = 320 × 320 × (208–240) with 7.7–6.7% partition oversampling, spatial resolution = 0.53 × 0.53 × 0.53 mm^3^ (without zero-filled interpolation), turbo factor = 52, echo train duration = 271 ms, 6/8 partial Fourier in the partition-encoding direction, parallel imaging (GRAPPA) acceleration rate = 2 in the phase-encoding direction, scan time = 7 min 10 s – 8 min 10 s depending on the head size.

In 19 out of the 34 healthy subjects, T1-weighted 2D TSE was also performed during the rescan session to provide an CMR imaging reference for assessing the inter-method agreement. Due to its relatively poor scan efficiency, the acquisition was prescribed only for three arterial segments that often present with ICAD in patients, including the distal basilar artery (BA), distal internal carotid artery (ICA) supraclinoid segment (C4), and proximal middle cerebral artery (MCA) M1 segment. Immediately after the 3D IVW CMR rescan, 3D images were reconstructed into three contiguous 2-mm-thick cross-sections at each of the three segments by an experienced CMR technologist using the multiplanar reconstruction (MPR) functionality available on the imaging console. A 2D TSE image was then acquired for each of these cross-sections with following imaging parameters: TR/TE = 800/12 ms, receiver bandwidth = 411 Hz/pixel, field of view = 170 × 170 mm^3^, matrix size = 320 × 320, spatial resolution = 0.53 × 0.53 mm^2^, slice thickness = 2 mm, turbo factor = 9, signal averages = 4, scan time per slice = 1 min 37 s.

### Image analysis

All images were transferred to a workstation (Syngo MultiModality Workplace, Siemens Healthineers). The scan and rescan 3D IVW CMR image sets were first co-registered using an image fusion functionality to account for head repositioning. At the same locations on both image sets, 5 following vessel segments were analyzed for each healthy subject: the distal BA, the distal vertebral artery (VA; V4), the distal ICA C4, the proximal MCA M1, and the proximal anterior cerebral artery (ACA) A1. Three contiguous cross-sections of 2-mm thickness were generated via MPR for each segment. For each patient, 3 contiguous cross-sections of 2-mm thickness centered at the thickest location of the most stenotic plaque were also generated via MPR.

**Fig. 1:**
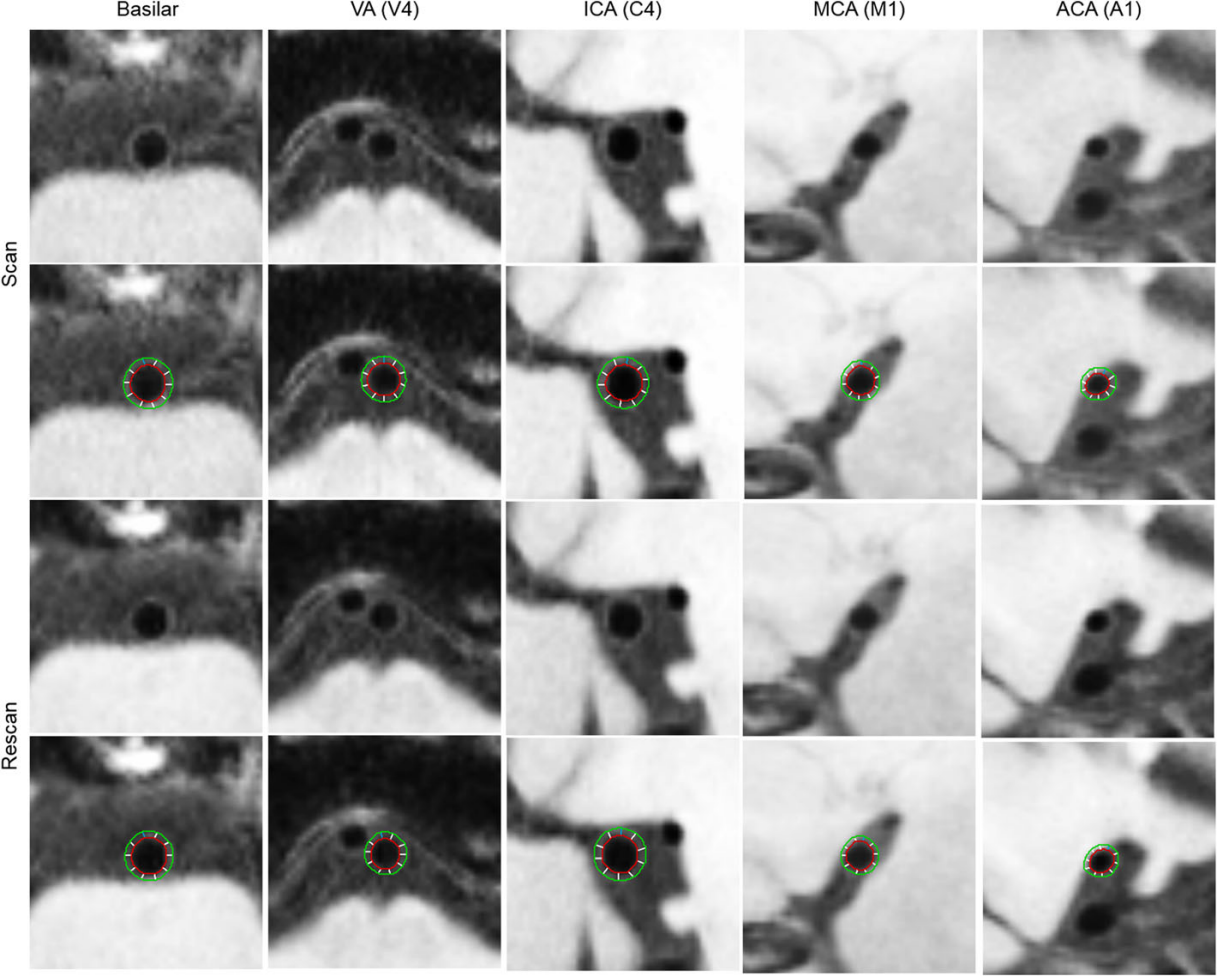
Representative images of scan and rescan analysis of lumen and outer wall boundaries for the five designated arterial segments: the distal basilar artery, the distal vertebral artery (VA (V4)), the distal internal carotid artery (ICA) supraclinoid segment (C4), the middle cerebral artery (MCA) M1 segment, and anterior cerebral artery (ACA) A1 segments, from a 55-year-old male healthy subject

All these reconstructed cross-sectional images and corresponding 2D TSE images underwent vessel wall and lumen dimension quantification using commercial software (VesselMass, Leiden University Medical Center, Leiden, the Netherlands). Each image was magnified 4–6 times with bilinear interpolation. Lumen and outer wall boundaries were traced manually along the interfaces between the lumen and wall and between the wall and surrounding tissue respectively, generating two contours (Fig. 1). When part of a boundary was invisible, the contour was completed to maintain the continuity of the vessel’s curvature [9]. The entire vessel wall region encased by the two contours were automatically divided into ten evenly spaced segments. The software generated the following measurements: the average and maximum wall thickness (i.e. the mean and maximum value of the ten distances between contours), the lumen area (i.e. the area inside the luminal contour), and the wall area (i.e. subtracting the inner contour area from the outer contour area). Additionally, normalized wall index was calculated as the ratio of the wall area to the outer contour area. Contouring and determination of the above wall and lumen dimensions for any 3 consecutive slices required a processing time of approximately 2.5 min per scan. For each vessel segment or plaque, the measured normalized wall index and mean/maximum wall thickness were, respectively, averaged over the three slices; lumen volume and wall volume were obtained by summing the area measurements of the three slices and multiplying by 2 mm.

Two readers (with 6-year and more than 10-year experience in vascular CMR imaging, respectively) independently performed above vessel wall and lumen measurements on the images from the first 3D IVW CMR scan. After two weeks, one of the readers performed a second-round measurement on the same data, and the other performed measurement on the images from the second scan followed by measurement another 2 weeks later for the 2D TSE scan when available.

### Statistical analysis

All statistical analyses were performed using SPSS (version 19.0, International Business Machines, Armonk, New York, USA) and R (version 3.4.1). Intra-class correlation coefficient (ICC) was obtained from a two-way random model with two raters for inter-rater reproducibility and a two-way mixed model with two raters for intra-rater and scan-rescan reproducibility. Confidence intervals for the overall ICC were calculated by bootstrap taking in account the correlation between segments in the same patient. An ICC value of less than 0.4 was considered poor agreement, a value of 0.4–0.75 was considered good agreement, and a value of 0.75 or greater was considered excellent agreement [26]. Bland-Altman analysis was also used to determine the scan-rescan, intra-, and inter-observer reproducibility of 3D IVW CMR as well as inter-method agreement between 3D IVW CMR and 2D TSE in quantifying vessel dimensions for volunteers.

In addition, the healthy cohort was further categorized by age into two groups, i.e. < 50 years and ≥50 years. All above reproducibility were determined for each group. Morphologic measurements averaged over the two readers were used to determine the differences between the two groups based on independent t-test. A two-tailed *P* value of 0.05 or less was considered to indicate a significant difference.

Based on the scan-rescan data analysis, the sample size required for each of dimension measurements to compare placebo and treatment group in a clinical trial with 80% of power at 5% significance level was calculated using a t-test with equal variances. It was assumed that the mean of the placebo group would be equal to the mean from the healthy subjects in our study and that the mean of the treatment group would be 5, 10, 15, and 20% different. The standard deviations for placebo and treatment groups were assumed to be equal and given by the subject variance estimated from a linear mixed model with subject as fixed effect and scan as random effect.

## Results

Motion-related vessel wall blurring was observed by the CMR technologist in either of the two 3D IVW CMR scans in 6 healthy subjects and 2 patients. Reacquisitions in these subjects were performed and yielded acceptable image quality in all but 2 healthy subjects (51 and 49 years) and 1 patient (61 years) who were excluded from image analysis. Hence, a total of 160 paired arterial segments from 32 healthy subjects (13: age < 50 years and 19: age ≥ 50 years) and 9 plaques (5 on MCA, 2 on BA, and 2 on VA) were available for reproducibility analysis; a total of 54 paired arterial segments from 18 healthy subjects were available for inter-method agreement analysis.

### Measurement reproducibility

**Fig. 2:**
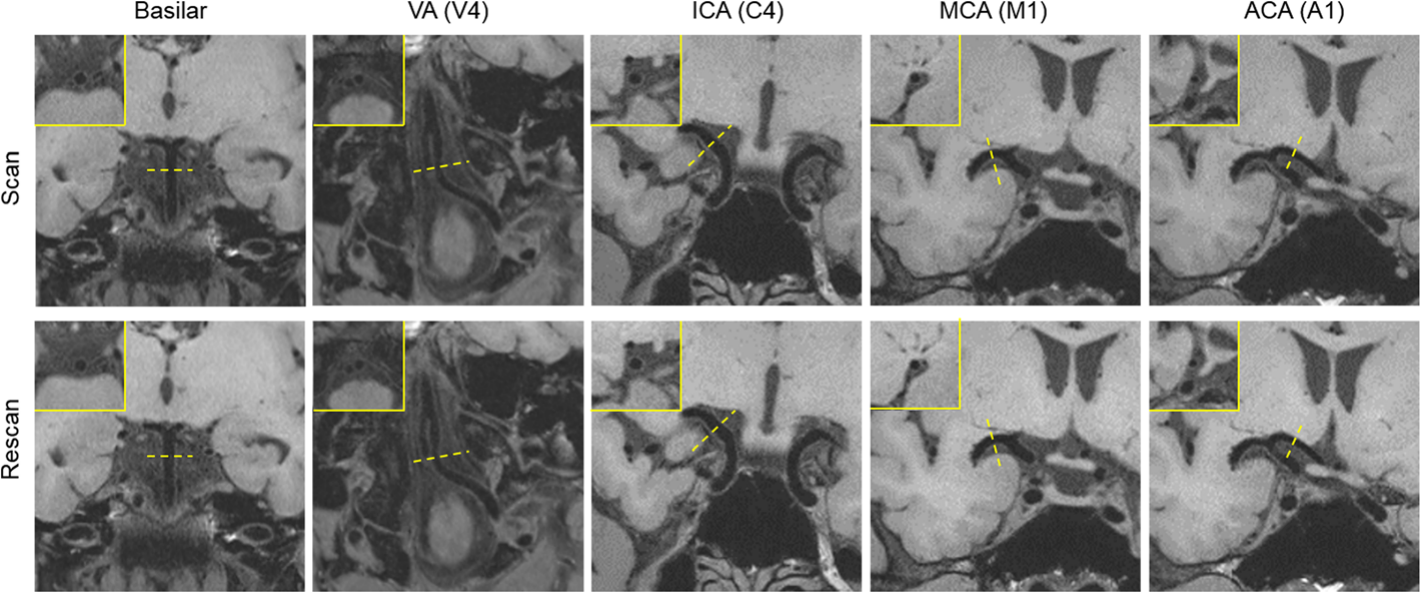
Representative 3D intracranial vessel wall MR images acquired in two scans from a 55-year-old male healthy subjects and reformatted cross-sections (upper left in the yellow box) for each designated arterial segment in the location indicated by the dashed lines. Both scans provide exquisite vessel wall depiction for the five designated arterial segments: the distal basilar artery, distal vertebral artery (VA (V4)), the distal internal carotid artery (ICA) supraclinoid segment (C4), the middle cerebral artery (MCA) M1 segment right after the trifurcation, and the anterior cerebral artery (ACA) A1 segments

**Fig. 3:**
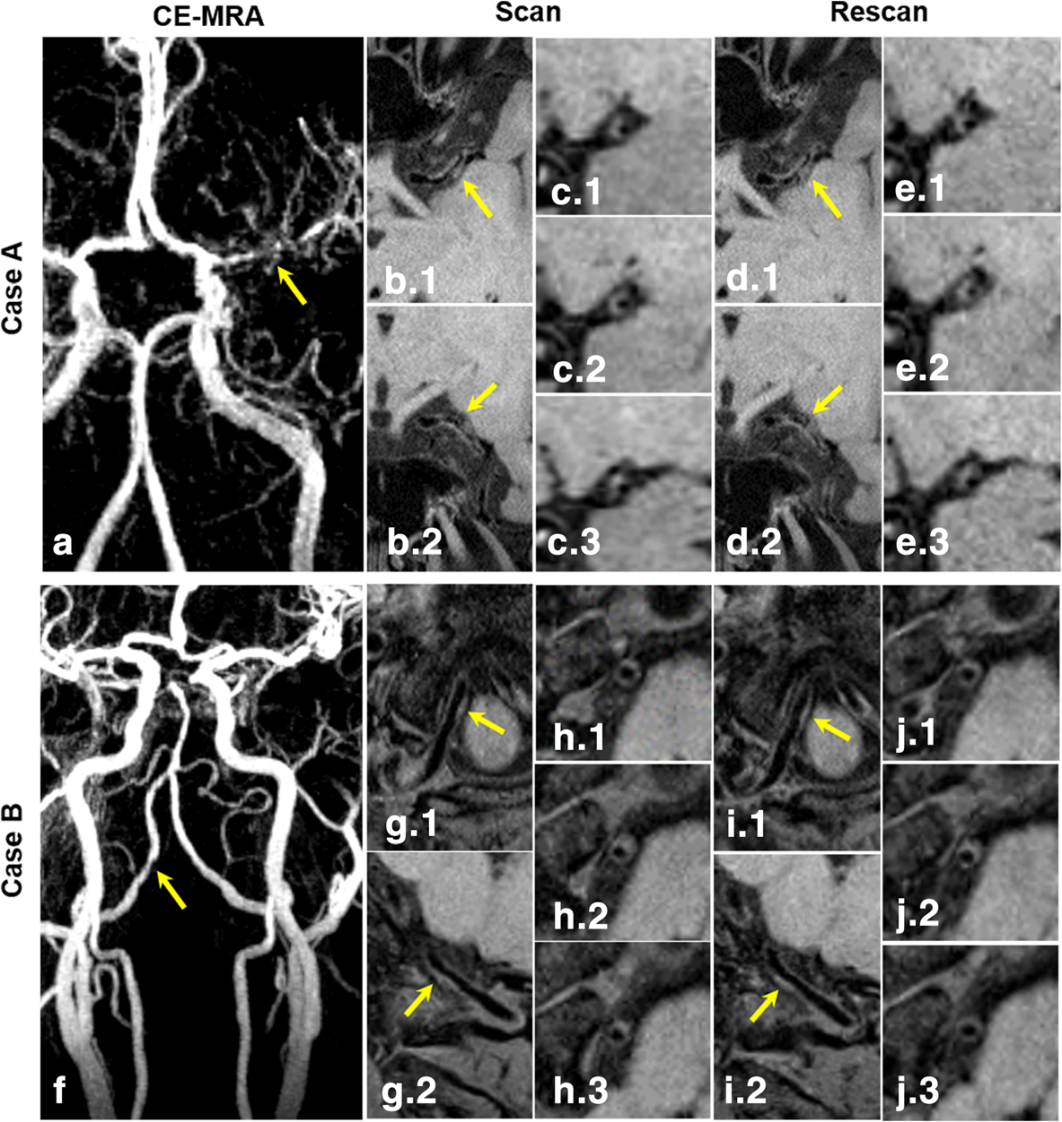
Two representative clinical cases imaged with 3D intracranial vessel wall (IVW) CMR. Contrast-enhanced MRA demonstrates a severe stenosis at the left middle cerebral artery (MCA) M1 segment (arrow in **a**) in a 42-year-old male patient and a moderate stenosis at the right vertebral artery (VA) (arrow in **f**) in a 48-year-old female patient. Reconstructed long-axis images from 3D whole-brain IVW scan and rescan reveal wall thickening at both stenoses (arrows in **b** and **d**, **g** and **i**). Reconstructed short-axis (cross-section) images for the MCA plaque (**c** and **e**) and VA plaque (**h** and **j**) demonstrate the eccentric wall thickening. Note that the delineation quality of these plaques from scan and rescan are visually comparable

3D IVW CMR provided visually consistent delineation of the vessel wall (Fig. 2) and plaques (Fig. 3) in both scans. In some plaques, high signal-intensity features were observed (Fig. 3 case A). For healthy subjects, morphologic measurements and corresponding ICC values, when combining all assessed segments, are summarized in Table 1 (Segment-based results are summarized in Additional file 1: Table S1). Each of the assessed morphologic indices had all ICCs greater than 0.75, indicating excellent reproducibility. More specifically, for the intra-observer reproducibility, all ICCs were equal to or greater than 0.93. For the scan-rescan and inter-observer reproducibility, all ICCs except for that for the inter-observer reproducibility on normalized wall index were equal to or greater than 0.83. For patients, vessel wall and lumen measurements at the most stenotic plaque and corresponding ICC values are summarized in Table 2. All ICCs except for that for the inter-observer reproducibility on maximum wall thickness (ICC = 0.87) were equal to or greater than 0.91, indicating excellent reproducibility.

**Table 1:** Vessel wall and lumen measurements averaged over all assessed segments and corresponding ICC values of inter-scan, intra-observer, and inter-observer reproducibilities of 3D intracranial vessel wall MR in healthy subjects

		inter-scan (*n* = 160)		Intra-observer (*n* = 160)		Inter-observer (*n* = 160)	
	1st scan, 1st observer, 1st measurement (mean ± SD)	2nd scan, 1st observer (mean ± SD)	ICC (95% CI)	1st scan, 1st observer, 2nd measurement (mean ± SD)	ICC (95% CI)	1st scan, 2nd observer (mean ± SD)	ICC (95% CI)
Lumen volume (mm^3^)	42.0 ± 16.4	42.9 ± 16.8	0.99 (0.98–0.99)	43.3 ± 16.6	0.99 (0.98–0.99)	40.4 ± 17.4	0.98 (0.97–0.99)
Wall volume (mm^3^)	49.7 ± 15.8	49.5 ± 16.6	0.98 (0.98–0.99)	50.1 ± 15.4	0.99 (0.98–0.99)	45.7 ± 17.2	0.93 (0.80–0.96)
Normalized wall index	0.55 ± 0.04	0.54 ± 0.05	0.87 (0.80–0.91)	0.54 ± 0.04	0.93 (0.89–0.95)	0.53 ± 0.06	0.76 (0.63–0.83)
Mean wall thickness (mm)	0.71 ± 0.11	0.70 ± 0.13	0.95 (0.93–0.96)	0.71 ± 0.11	0.96 (0.95–0.97)	0.67 ± 0.14	0.83 (0.67–0.89)
Maximum wall thickness (mm)	0.86 ± 0.12	0.85 ± 0.15	0.93 (0.90–0.95)	0.86 ± 0.13	0.95 (0.93–0.96)	0.85 ± 0.18	0.84 (0.78–0.88)

*SD* standard deviation, *ICC* intra-class correlation coefficient, *CI* confidence intervals

**Table 2:** Vessel wall and lumen measurements at the most stenotic plaques and corresponding ICC values of inter-scan, intra-observer, and inter-observer reproducibilities of 3D intracranial vessel wall MR in patients

		Inter-scan (*n* = 9)		Intra-observer (*n* = 9)		Inter-observer (*n* = 9)	
	1st scan, 1st observer, 1st measurement (mean ± SD)	2nd scan, 1st observer (mean ± SD)	ICC (95% CI)	1st scan, 1st observer, 2nd measurement (mean ± SD)	ICC (95% CI)	1st scan, 2nd observer (mean ± SD)	ICC (95% CI)
Lumen volume (mm^3^)	25.1 ± 18.2	26.57 ± 21.33	0.99 (0.94–0.99)	25.7 ± 17.6	0.99 (0.99–0.99)	23.7 ± 18.0	0.99 (0.96–0.99)
Wall volume (mm^3^)	54.2 ± 32.6	55.36 ± 31.82	0.99 (0.97–0.99)	54.5 ± 29.4	0.99 (0.96–0.99)	50.2± 26.3	0.98 (0.90–0.99)
Normalized wall index	0.69 ± 0.08	0.69 ± 0.10	0.93 (0.66–0.98)	0.69 ± 0.09	0.98 (0.89–0.99)	0.69 ± 0.10	0.92 (0.65–0.98)
Mean wall thickness (mm)	0.90 ± 0.29	0.92 ± 0.28	0.97 (0.89–0.99)	0.91 ± 0.25	0.97 (0.89–0.99)	0.88 ± 0.25	0.97 (0.86–0.99)
Maximum wall thickness (mm)	1.35 ± 0.37	1.39 ± 0.31	0.91 (0.59–0.98)	1.44 ± 0.37	0.94 (0.76–0.99)	1.25 ± 0.29	0.87 (0.49–0.97)

*SD* standard deviation, *ICC* intra-class correlation coefficient, *CI* confidence intervals

**Fig. 4:**
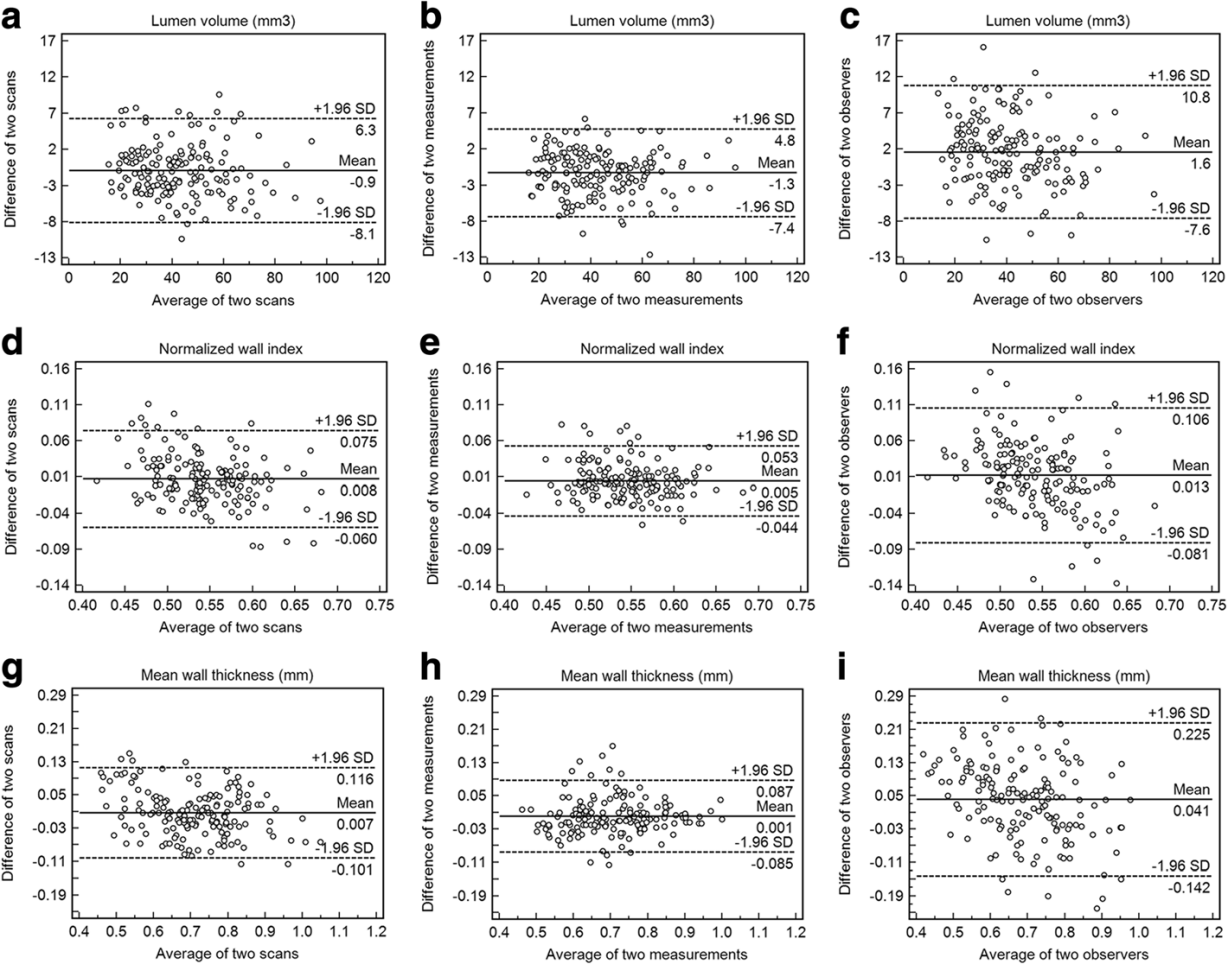
Bland-Altman plots for lumen volume (**a** inter-scan, **b** intra-observer, **c** inter-observer), normalized wall index (**d** inter-scan, **e** intra-observer, **f** inter-observer), and mean wall thickness (**g** inter-scan, **h** intra-observer, **i** inter-observer). The solid lines represent the mean difference, and the dashed lines indicate the 95% limits of agreement. SD = standard deviation

The Bland-Altman plots for all arterial segments of healthy subjects are shown in Fig. 4 for lumen volume, normalized wall index, and mean wall thickness, respectively. Random error scattering patterns and independence of the difference on the mean value were observed.

**Fig. 5:**
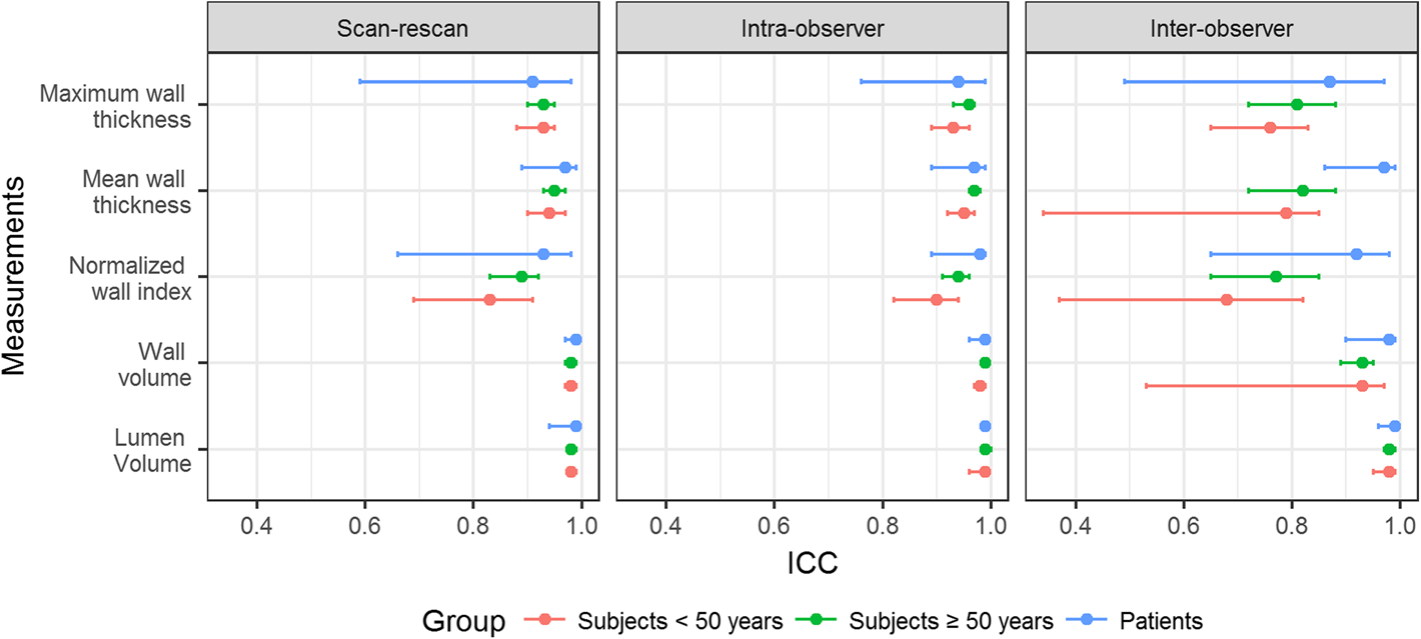
The comparison of ICCs (95% CI) for all vessel wall and lumen measurements among patients and different age groups of healthy subjects. ICC = intra-class correlation coefficient; CI = confidence interval

All ICCs of the healthy subgroup age ≥ 50 years were equal to or higher than that of the < 50 years subgroups, but in most cases, were lower than that of patients (Fig. 5). As shown in Table 3, there were no significant difference in lumen or wall volume between the two age groups. However, normalized wall index and mean and maximum wall thickness were significantly larger in the age group of ≥50 years (*P* ≤ 0.05).

**Table 3:** The comparison for the vessel wall and lumen measurements averaged over two observers between different age subgroups. Data are presented as means ± standard deviations

Age group	Lumen volume (mm^3^)	Wall volume (mm^3^)	Normalized wall index^a^	Mean wall thickness (mm)^a^	Maximum wall thickness (mm)^a^
≥50 years	41.2 ± 16.7	50.5 ± 16.0	0.55 ± 0.06	0.69 ± 0.14	0.88 ± 0.18
< 50 years	47.7 ± 16.1	48.9 ± 15.5	0.51 ± 0.04	0.63 ± 0.14	0.80 ± 0.17
*P* value	0.23	0.61	< 0.001	0.05	0.03

^a^denotes statistical significance

### Inter-method agreement

**Fig. 6:**
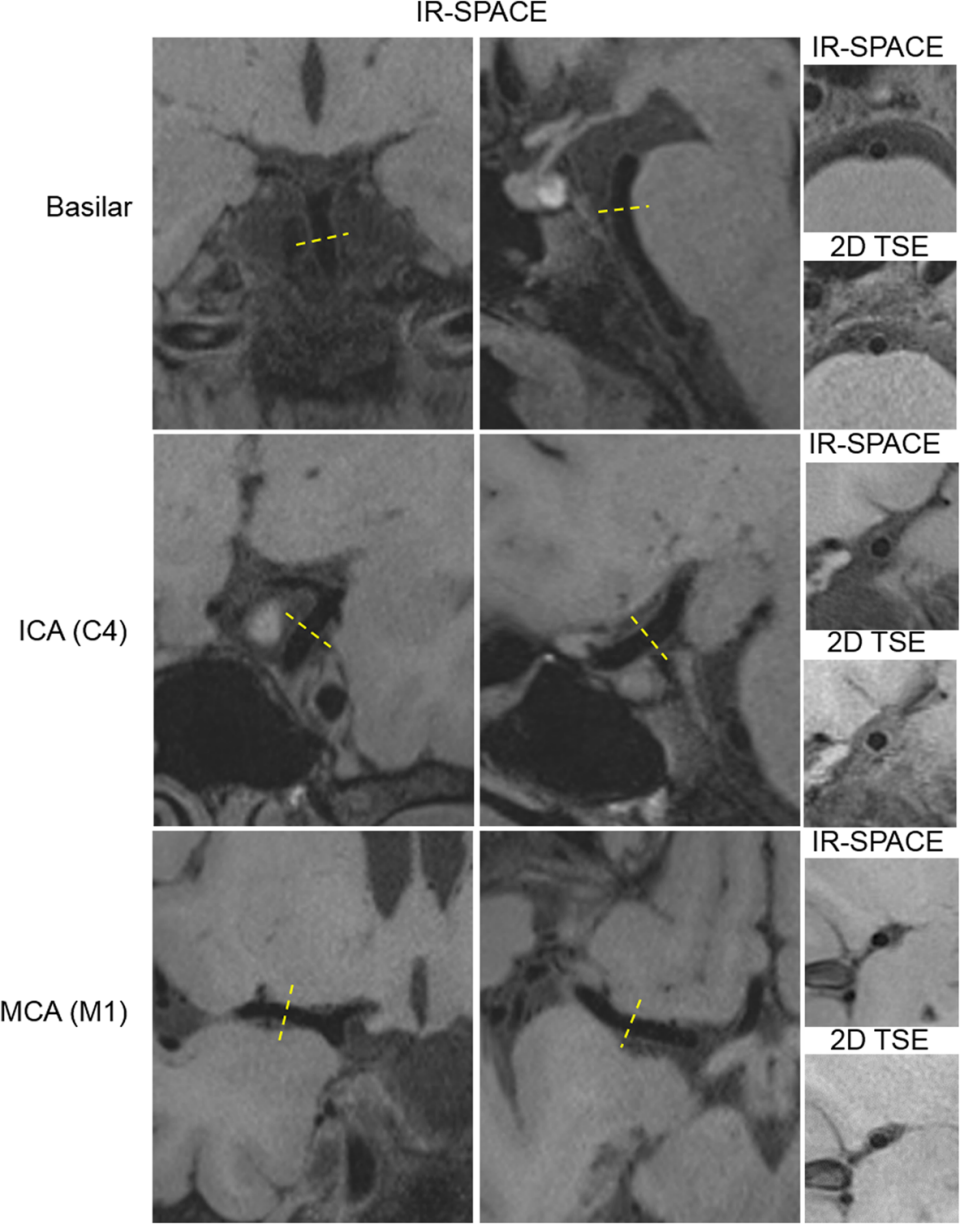
Representative 3D intracranial vessel wall CMR images (left and middle columns: two different long axis views; right column: reformatted cross-sections in the location indicated by the dashed lines in long axis views) and corresponding 2D intracranial vessel wall CMR images in a 31-year-old male volunteer. 3D intracranial vessel wall CMR and 2D TSE provide comparable vessel wall delineation for the three arterial segments: distal basilar artery, distal internal carotid artery (ICA) supraclinoid segment (C4), and the proximal M1 segment of middle cerebral artery (MCA). All cross-sectional images reformatted from 3D IVW MR are of 2-mm thickness, matched with that on 2D TSE images

**Fig. 7:**
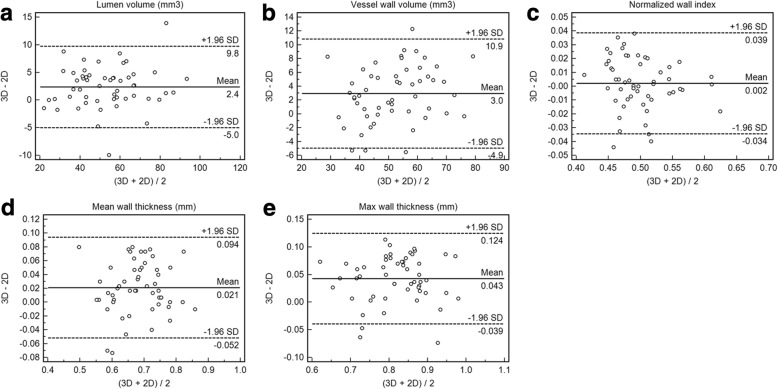
Bland-Altman plots of the difference versus mean for the 3D and 2D intracranial vessel wall CMR paired intracranial vessel wall and lumen measurements show good agreement with a small bias of 2.4 mm^3^, 3.0 mm^3^, 0.002, 0.02 mm, and 0.04 mm for lumen volume (**a**), vessel wall volume (**b**), normalized wall index (**c**), mean wall thickness (**d**) and maximum wall thickness (**e**), respectively. The solid lines represent the mean difference, and the dashed lines indicate the 95% limits of agreement. SD = standard deviation

Figure 6 shows representative 3D IVW images and reformatted vessel wall cross-sections as well as slice thickness- and location-matched 2D TSE images for three arterial segments. The two acquisition methods provided visually comparable vessel wall delineation. When all three segments were evaluated together, all paired morphologic measurements exhibited an excellent agreement as indicated by an ICC with 95% CI of 0.98 (0.95–0.99), 0.96 (0.84–0.98), 0.96 (0.92–0.97), 0.92 (0.82–0.96), and 0.88 (0.32–0.96) for lumen volume, wall volume, normalized wall index, mean wall thickness, and maximum wall thickness, respectively. Segment-based morphologic measurements and corresponding ICC values for 3D and 2D IVW CMR are summarized in Additional file 1: Table S2. The differences between 3D and 2D IVW CMR and the mean values with limits of agreement for all segments are illustrated in Bland-Altman plots (Fig. 7). The mean differences between 3D and 2D IVW CMR were 2.4 mm^3^ for lumen volume, 3.0 mm^3^ for vessel wall volume, 0.002 for normalized wall index, 0.02 mm for mean wall thickness, and 0.04 mm for maximum wall thickness. Bland-Altman analysis demonstrated good agreement with small bias between the two techniques.

### Sample size

Table 4 shows sample size required to compare placebo and treatment group for all measures means considering MCA segment with 80% of power at 5% significance level using a t-test for two independent samples with equal variance. The sample sizes required for the measures in other segments are also presented in Additional file 1: Table S3. Normalized wall index requires the smallest sample size while lumen volume requires the highest sample size to compare two groups. The large-sized segments (ICA, VA, and BA) requires the smaller sample size than small-sized segments (MCA and ACA).

**Table 4:** Sample Sizes per Group for differences of 5%, 10%, 15%, and 20% from placebo group mean estimated in the middle cerebral artery segment based on the inter-scan analysis

	Placebo group (mean ± SD)	Sample size			
		5%	10%	15%	20%
Lumen volume (mm^3^)	38.9 ± 8.9	329	83	38	22
Wall volume (mm^3^)	41.1 ± 7.6	215	55	25	15
Normalized wall index	0.52 ± 0.05	60	16	8	5
Mean wall thickness (mm)	0.63 ± 0.08	103	27	13	8
Maximum wall thickness (mm)	0.77 ± 0.1	107	28	13	8

*SD* standard deviation

## Discussion

A non-invasive imaging method for reliably quantifying longitudinal morphologic changes in ICAD is potentially useful in medical management or drug development. High-resolution black-blood 2D CMR, traditionally used for ICAD imaging, has been shown to be a morphology-probing tool with good intra- and inter-observer agreement [27] as well as low scan-rescan variability [28]. With aforementioned technical advantages that are more relevant to vessel wall morphologic assessments, 3D IVW CMR has increasingly been advocated as a non-invasive imaging modality for ICAD research [9–15]. However, its applicability in longitudinal imaging evaluations has yet to be established. Thus, the present study sought to conduct a comprehensive investigation on its reliability in the quantification of intracranial vessel dimensions.

Scan-rescan reproducibility is a paramount requirement for an imaging modality to be used for serial examinations. Our results showed excellent scan-rescan reproducibility in measuring the dimensions of major intracranial arterial segments for healthy subjects and plaques for patients with all ICCs ≥0.87 and 0.91, respectively. A previous study on using 2D IVW CMR for evaluating MCA lumen and plaque area/volume showed better ICCs (0.97 or higher) [28]. This is likely because the 3D technique is more susceptible to any errors caused by, for example, image registration, reformation, and vessel wall contouring, particularly in healthy subjects where the vessel wall is thinner. A more recent population-based study has reported considerably lower reproducibility in wall volume, normalized wall index, and mean wall thickness for a 3D IVW CMR sequence [25]. One of major possible reasons for the better performance of the whole-brain IVW CMR sequence in our study is that two repeat scans were in the same imaging session in healthy volunteers or days apart in patients.. Clearly this same-session investigation strategy for healthy subjects only reveals the scan-rescan repeatability instead of the true longitudinal repeatability of a technique, but has commonly been used in many previous studies [26, 28, 29]. Nevertheless, our findings suggest the possibility for reliable serial examination of IVW using the 3D whole-brain IVW CMR technique as previous carotid studies did [26, 29, 30].

Our study also revealed excellent intra- and inter-observer reproducibility in quantifying intracranial vessel dimensions. In general, all ICCs were better than those reported by the recent population-based study whereby a slab-selective 3D IVW CMR sequence was used [25]. Our evaluations were focused on a recently developed whole-brain vessel wall CMR imaging method because of its several technical advantages over other existing slab-selective 3D IVW imaging techniques [18]. A noteworthy feature is its more superior delineation of the outer vessel wall boundary due to an improved signal suppression in surrounding CSF [18]. Additionally, relatively short echo time due to the use of a non-selective excitation radio-frequency pulse may contribute to better overall image SNR. Hence, quantification of vessel area, wall area, and wall thickness would potentially be more accurate.

As part of reliability analysis, the present study investigated the inter-method agreement between 3D IVW CMR and conventionally used 2D TSE. The lumen and vessel wall volume and normalized wall index measured from 3D IVW CMR showed excellent accordance with those measured from conventional 2D TSE (ICC > 0.96) despite potential registration errors. This corroborates the findings reported in the previous study [9]. The ICCs of mean and maximum wall thickness, particularly the latter, were slightly lower, which could be explained by the fact that these measurements are more prone to outliers [26]. Nevertheless, they generally showed excellent agreement between the two techniques. While the accuracy of the 3D technique is questionable due to the lack of histology validation, our finding suggests that this technique is at least comparable to the 2D technique and can be utilized as a more time-efficient ICAD imaging method. Given the much higher and isotropic spatial resolution and flexibility in image reformation with 3D imaging, the geometry of small lesions from the tortuous intracranial arteries would, in theory, be quantified more accurately.

In general, relatively large-sized segments exhibited higher reproducibility and smaller sample size required than small-sized segments, and the age group of ≥50 years demonstrated equal or higher reproducibility than the younger group. Additionally, the patient group demonstrated an even better reproducibility than the age group of ≥50. This is perhaps explained by the thicker vessel wall in large-sized segments and in older subjects or patients that is favorable for morphologic quantification. Our results did show that normalized wall index and mean and maximum wall thickness were significantly larger in the age group of ≥50 years versus the younger group and in the patient group versus the healthy group. The limit in spatial resolution and associated errors in image registration and contouring are thought of as major factors influencing the measurement consistency. It is noteworthy that in clinical patients who have ICAD lesions or dramatically thickened vessel wall, such an effect might be alleviated. Additionally, 0.5 mm spatial resolution provided by the whole-brain IVW CMR technique is currently the best choice given the trade-off between imaging time and diagnostic quality as recommended [31].

With such high reproducibility of vessel dimension measurements, whole-brain IVW CMR imaging can potentially be translated into research and clinical applications for monitoring disease progression and therapeutic response. More importantly, higher inter-scan reproducibility promises fewer participants for therapeutic trial enrollment and reduced cost. Sample sizes for MCA segment presented are higher than Zhang et al. [28] because they based their calculations on the standard deviation between scans while we used standard deviations resulting from the total variance decreased by the variance between scans as Mihai et al. [32].

There are limitations with this work. First, we focused reliability analyses on healthy subjects and only 9 patients were included. Despite relatively large vessel wall dimension in ICAD patients which favors morphologic measurement, reproducibility could be compromised by, for example, reduced image quality due to motion. Reproducibility studies based on healthy subjects have commonly been investigated in the field of vessel wall CMR imaging [9, 26, 29, 33]. The results from this type of study may provide indication of the technical performance in general populations as well as insights into planning future studies on clinical patients. Second, the 3D technique is still susceptible to motion artifacts which occurred in 6 out of 34 healthy subjects and in 1 out of 10 patients. Four of the 6 healthy subjects were still eligible for analysis as reacquisition was of acceptable image quality. Hence, our conclusion holds valid when only considering cases with acceptable diagnostic quality. Further improvement in motion resistance is clearly necessary to foster the technique’s clinical reliability. Third, the scan and rescan were performed on the same CMR scanner with the same CMR technologist. Thus, we could not estimate any variation caused by imaging scanners or between MR technologists.

## Conclusion

In conclusion, whole-brain 3D IVW CMR is a reliable CMR imaging method for the quantification of intracranial vessel dimensions and could potentially be useful for monitoring plaque progression and regression.

## Additional file

Additional file 1: **Table S1.** Segment-based vessel wall and lumen measurements and corresponding ICC values of inter-scan, intra-observer, and inter-observer reproducibility of 3D intracranial vessel wall CMR in healthy subjects. **Table S2.** Agreement between 3D and 2D intracranial vessel wall CMR in the quantification of vessel dimensions. All vessel wall and lumen measurements are presented as means ± standard deviations. **Table S3.** Sample Sizes per Group for differences of 5%, 10%, 15%, and 20% from placebo group mean estimated based on the inter-scan analysis. (DOCX 27 kb)

## Abbreviations

2D: Two-dimensional
3D: Three-dimensional
A1: Proximal ACA
ACA: Anterior cerebral artery
BA: Basilar artery
C4: Distal ICA supraclinoid segment
CMR: Cardiovascular magnetic resonance
CSF: Cerebrospinal fluid
ICA: Internal carotid artery
ICAD: Intracranial atherosclerotic disease
ICC: Intra-class correlation coefficient
IR: Inversion-recovery
IVW: Intracranial vessel wall
M1: Proximal middle cerebral artery
MCA: Middle cerebral artery
MPR: Multiplanar reconstruction
SNR: Signal-to-noise ratio
SPACE: Sampling Perfection with Application-optimized Contrast using different flip angle Evolutions
TE: Echo time
TR: Repetition time
TSE: Turbo spin-echo
V4: Distal vertebral artery
VA: vertebral artery
