# Supplementary material for: 3D whole-brain vessel wall cardiovascular magnetic resonance imaging: a study on the reliability in the quantification of intracranial vessel dimensions
Source: J Cardiovasc Magn Reson. 2018 Jun 14;20:39. doi: 10.1186/s12968-018-0453-z (PMC6000985; doi:10.1186/s12968-018-0453-z)
Supplement: Supplementary file 1 — Table S1. Segment-based vessel wall and lumen measurements and corresponding ICC values of inter-scan, intra-observer, and inter-observer reproducibility of 3D intracranial vessel wall CMR in healthy subjects. Table S2. Agreement between 3D and 2D intracranial vessel wall CMR in the quantification of vessel dimensions. All vessel wall and lumen measurements are presented as means ± standard deviations. Table S3. Sample Sizes per Group for differences of 5%, 10%, 15%, and 20% from placebo group mean estimated based on the inter-scan analysis. (DOCX 27 kb) [file 12968_2018_453_MOESM1_ESM.docx]

**Table S1** Segment-based vessel wall and lumen measurements and corresponding ICC values of inter-scan, intra-observer, and inter-observer reproducibility of 3D intracranial vessel wall MR in healthy volunteers.

|  |  | Inter-scan (n=32) | |  | Intra-observer (n=32) | |  | Inter-observer (n=32) | |
| --- | --- | --- | --- | --- | --- | --- | --- | --- | --- |
|  | 1^st^ scan, 1^st^ observer, 1^st^ measurement  (mean ± SD) | 2^nd^ scan, 1^st^ observer  (mean ± SD) | ICC  (95% CI) |  | 1^st^ scan, 1^st^ observer, 2^nd^ measurement  (mean ± SD)^a^ | ICC  (95% CI) |  | 1^st^ scan, 2^nd^ observer  (mean ± SD) | ICC  (95% CI) |
| ICA |  |  |  |  |  |  |  |  |  |
| Lumen volume (mm^3^) | 63.58 ± 13.81 | 64.70 ± 14.38 | 0.98  (0.96-0.99) |  | 65.06 ± 13.40 | 0.99  (0.97-0.99) |  | 63.23 ± 14.11 | 0.98  (0.96-0.99) |
| Wall volume (mm^3^) | 68.11 ± 9.98 | 68.52 ± 10.53 | 0.92  (0.83-0.96) |  | 67.65 ± 11.54 | 0.96  (0.92-0.98) |  | 63.58 ± 12.22 | 0.81  (0.54-0.91) |
| Normalized wall index | 0.52 ± 0.03 | 0.52 ± 0.04 | 0.91  (0.81-0.95) |  | 0.51 ± 0.04 | 0.89  (0.76-0.95) |  | 0.50 ± 0.05 | 0.74  (0.46-0.88) |
| Mean wall thickness (mm) | 0.81 ± 0.07 | 0.81 ± 0.08 | 0.86  (0.72-0.93) |  | 0.80 ± 0.09 | 0.91  (0.81-0.95) |  | 0.76 ± 0.11 | 0.65  (0.28-0.83) |
| Maximum wall thickness (mm) | 0.97 ± 0.08 | 0.98 ± 0.10 | 0.83  (0.65-0.92) |  | 0.96 ± 0.11 | 0.86  (0.72-0.93) |  | 0.97 ± 0.15 | 0.67  (0.31-0.84) |
| MCA |  |  |  |  |  |  |  |  |  |
| Lumen volume (mm^3^) | 38.09 ± 8.86 | 39.73 ± 8.89 | 0.96  (0.90-0.98) |  | 39.24 ± 9.72 | 0.94  (0.89-0.97) |  | 34.34 ± 9.36 | 0.83  (0.56-0.92) |
| Wall volume (mm^3^) | 41.76 ± 7.29 | 40.37 ± 7.83 | 0.90  (0.79-0.95) |  | 43.56 ± 6.99 | 0.93  (0.81-0.97) |  | 36.96 ± 9.61 | 0.73  (0.31-0.88) |
| Normalized wall index | 0.53 ± 0.04 | 0.51 ± 0.05 | 0.81  (0.53-0.91) |  | 0.53 ± 0.04 | 0.92  (0.83-0.96) |  | 0.52 ± 0.06 | 0.66  (0.31-0.84) |
| Mean wall thickness (mm) | 0.64 ± 0.06 | 0.61 ± 0.09 | 0.81  (0.59-0.91) |  | 0.66 ± 0.07 | 0.89  (0.77-0.95) |  | 0.60 ± 0.11 | 0.66  (0.29-0.83) |
| Maximum wall thickness (mm) | 0.79 ± 0.08 | 0.75 ± 0.10 | 0.78  (0.52-0.90) |  | 0.80 ± 0.07 | 0.92  (0.83-0.96) |  | 0.76 ± 0.14 | 0.66  (0.31-0.83) |
| ACA |  |  |  |  |  |  |  |  |  |
| Lumen volume (mm^3^) | 24.66 ± 5.14 | 24.92 ± 6.29 | 0.88  (0.75-0.94) |  | 25.81 ± 5.81 | 0.90  (0.79-0.95) |  | 21.84 ± 5.77 | 0.80  (0.40-0.92) |
| Wall volume (mm^3^) | 30.87 ± 4.57 | 29.76 ± 5.42 | 0.89  (0.77-0.95) |  | 32.51 ± 5.55 | 0.87  (0.69-0.94) |  | 27.31 ± 7.85 | 0.66  (0.25-0.84) |
| Normalized wall index | 0.56 ± 0.04 | 0.55 ± 0.07 | 0.76  (0.51-0.88) |  | 0.56 ± 0.04 | 0.95  (0.89-0.97) |  | 0.55 ± 0.06 | 0.69  (0.36-0.85) |
| Mean wall thickness (mm) | 0.58 ± 0.05 | 0.56 ± 0.09 | 0.80  (0.59-0.90) |  | 0.59 ± 0.06 | 0.91  (0.80-0.96) |  | 0.54 ± 0.10 | 0.61  (0.21-0.81) |
| Maximum wall thickness (mm) | 0.72 ± 0.07 | 0.69 ± 0.10 | 0.77  (0.53-0.89) |  | 0.74 ± 0.07 | 0.88  (0.71-0.94) |  | 0.69 ± 0.12 | 0.74  (0.47-0.87) |
| VA |  |  |  |  |  |  |  |  |  |
| Lumen volume (mm^3^) | 39.40 ± 11.20 | 40.30 ± 11.33 | 0.96  (0.92-0.98) |  | 40.42 ± 10.69 | 0.99  (0.97-0.99) |  | 39.63 ± 11.53 | 0.97  (0.93-0.98) |
| Wall volume (mm^3^) | 54.33 ± 14.10 | 54.68 ± 14.70 | 0.98  (0.97-0.99) |  | 53.96 ± 14.59 | 0.99  (0.97-0.99) |  | 50.71 ± 15.83 | 0.87  (0.73-0.94) |
| Normalized wall index | 0.58 ± 0.03 | 0.58 ± 0.03 | 0.88  (0.75-0.94) |  | 0.57 ± 0.04 | 0.87  (0.73-0.94) |  | 0.56 ± 0.05 | 0.59  (0.18-0.80) |
| Mean wall thickness (mm) | 0.78 ± 0.11 | 0.78 ± 0.11 | 0.96  (0.92-0.98) |  | 0.77 ± 0.12 | 0.96  (0.91-0.98) |  | 0.74 ± 0.14 | 0.69  (0.38-0.85) |
| Maximum wall thickness (mm) | 0.93 ± 0.12 | 0.94 ± 0.12 | 0.91  (0.82-0.96) |  | 0.92 ± 0.14 | 0.94  (0.88-0.97) |  | 0.93 ± 0.19 | 0.72  (0.42-0.86) |
| BA |  |  |  |  |  |  |  |  |  |
| Lumen volume (mm^3^) | 44.47 ± 11.95 | 44.98 ± 12.13 | 0.98  (0.97-0.99) |  | 46.04 ± 12.54 | 0.98  (0.95-0.99) |  | 43.18 ± 12.46 | 0.97  (0.94-0.99) |
| Wall volume (mm^3^) | 53.21 ± 9.42 | 54.10 ± 9.12 | 0.96  (0.91-0.98) |  | 52.75 ± 9.25 | 0.98  (0.96-0.99) |  | 49.90 ± 12.59 | 0.89  (0.73-0.95) |
| Normalized wall index | 0.55 ± 0.04 | 0.55 ± 0.04 | 0.94  (0.88-0.97) |  | 0.54 ± 0.04 | 0.92  (0.83-0.96) |  | 0.54 ± 0.05 | 0.85  (0.70-0.93) |
| Mean wall thickness (mm) | 0.74 ± 0.06 | 0.75 ± 0.06 | 0.87  (0.73-0.93) |  | 0.73 ± 0.06 | 0.90  (0.79-0.95) |  | 0.71 ± 0.11 | 0.77  (0.50-0.89) |
| Maximum wall thickness (mm) | 0.89 ± 0.06 | 0.90 ± 0.06 | 0.86  (0.72-0.93) |  | 0.88 ± 0.08 | 0.82  (0.63-0.91) |  | 0.89 ± 0.14 | 0.75  (0.49-0.88) |

Note: SD, standard deviation; ICC, intra-class correlation coefficient; CI, confidence intervals; ICA, internal carotid artery; MCA, middle cerebral artery; ACA, anterior cerebral artery; VA, vertebral artery; BA, basilar artery.

**Table S2** Agreement between 3D and 2D intracranial vessel wall MR in the quantification of vessel dimensions. All vessel wall and lumen measurements are presented as means ± standard deviations.

|  |  | Lumen volume (mm^3^) | Wall volume (mm^3^) | Normalized wall index | Mean wall thickness (mm) | Maximum wall thickness (mm) |
| --- | --- | --- | --- | --- | --- | --- |
| All segments | 3D | 52.02 ± 16.43 | 50.20 ± 11.33 | 0.50 ± 0.04 | 0.67 ± 0.07 | 0.80 ± 0.08 |
|  | 2D | 54.41 ± 16.83 | 53.16 ± 12.38 | 0.50 ± 0.04 | 0.69 ± 0.07 | 0.84 ± 0.08 |
|  | ICC (95% CI) | 0.98 (0.95-0.99) | 0.96 (0.84-0.98) | 0.96 (0.92-0.97) | 0.92 (0.82-0.96) | 0.88 (0.32-0.96) |
| ICA | 3D | 68.55 ± 11.12 | 60.63 ± 7.94 | 0.47 ± 0.02 | 0.71 ± 0.06 | 0.85 ± 0.06 |
|  | 2D | 71.59 ± 11.12 | 65.75 ± 6.83 | 0.48 ± 0.02 | 0.75 ± 0.04 | 0.90 ± 0.04 |
|  | ICC (95% CI) | 0.95 (0.76-0.99) | 0.85 (0.14-0.97) | 0.86 (0.59-0.95) | 0.76 (0.13-0.93) | 0.62 (0.24-0.88) |
| MCA | 3D | 42.33 ± 9.41 | 40.29 ± 6.41 | 0.49 ± 0.03 | 0.60 ± 0.05 | 0.71 ± 0.05 |
|  | 2D | 45.77 ± 8.68 | 42.48 ± 7.49 | 0.48 ± 0.02 | 0.60 ± 0.04 | 0.75 ± 0.06 |
|  | ICC (95% CI) | 0.95 (0.24-0.99) | 0.87 (0.63-0.95) | 0.84 (0.58-0.94) | 0.78 (0.41-0.92) | 0.75 (0.21-0.91) |
| BA | 3D | 45.19 ± 13.85 | 49.69 ± 8.77 | 0.53 ± 0.05 | 0.70 ± 0.06 | 0.83 ± 0.06 |
|  | 2D | 45.88 ± 14.71 | 51.25 ± 9.08 | 0.54 ± 0.04 | 0.72 ± 0.05 | 0.86 ± 0.05 |
|  | ICC (95% CI) | 0.98 (0.94-0.99) | 0.95 (0.86-0.98) | 0.98 (0.96-0.99) | 0.93 (0.79-0.98) | 0.82 (0.07-0.95) |

Note: SD, standard deviation; ICC, intra-class correlation coefficient; CI, confidence intervals; ICA, internal carotid artery; MCA, middle cerebral artery; BA, basilar artery.

**Table S3** Sample Sizes per Group for differences of 5%, 10%, 15%, and 20% from placebo group mean estimated based on the inter-scan analysis.

|  | Placebo group  (mean ± SD) | Sample size | | | |
| --- | --- | --- | --- | --- | --- |
|  |  | 5% | 10% | 15% | 20% |
| ICA |  |  |  |  |  |
| Lumen volume (mm^3^) | 64.14 ± 14.1 | 305 | 77 | 35 | 20 |
| Wall volume (mm^3^) | 68.31 ± 10.26 | 143 | 37 | 17 | 10 |
| Normalized wall index | 0.52 ± 0.04 | 39 | 11 | 6 | 4 |
| Mean wall thickness (mm) | 0.81 ± 0.08 | 63 | 17 | 8 | 5 |
| Maximum wall thickness (mm) | 0.97 ± 0.1 | 68 | 18 | 9 | 6 |
| MCA |  |  |  |  |  |
| Lumen volume (mm^3^) | 38.91 ± 8.88 | 329 | 83 | 38 | 22 |
| Wall volume (mm^3^) | 41.07 ± 7.57 | 215 | 55 | 25 | 15 |
| Normalized wall index | 0.52 ± 0.05 | 60 | 16 | 8 | 5 |
| Mean wall thickness (mm) | 0.63 ± 0.08 | 103 | 27 | 13 | 8 |
| Maximum wall thickness (mm) | 0.77 ± 0.1 | 107 | 28 | 13 | 8 |
| ACA |  |  |  |  |  |
| Lumen volume (mm^3^) | 24.79 ± 5.74 | 338 | 86 | 39 | 23 |
| Wall volume (mm^3^) | 30.31 ± 5.02 | 174 | 45 | 21 | 12 |
| Normalized wall index | 0.55 ± 0.06 | 76 | 20 | 10 | 6 |
| Mean wall thickness (mm) | 0.57 ± 0.08 | 125 | 32 | 15 | 9 |
| Maximum wall thickness (mm) | 0.7 ± 0.09 | 105 | 27 | 13 | 8 |
| VA |  |  |  |  |  |
| Lumen volume (mm^3^) | 39.85 ± 11.27 | 504 | 127 | 57 | 33 |
| Wall volume (mm^3^) | 54.51 ± 14.4 | 440 | 111 | 50 | 29 |
| Normalized wall index | 0.58 ± 0.04 | 31 | 9 | 5 | 4 |
| Mean wall thickness (mm) | 0.78 ± 0.11 | 126 | 33 | 15 | 9 |
| Maximum wall thickness (mm) | 0.94 ± 0.12 | 104 | 27 | 13 | 8 |
| BA |  |  |  |  |  |
| Lumen volume (mm^3^) | 44.72 ± 12.04 | 457 | 115 | 52 | 30 |
| Wall volume (mm^3^) | 53.65 ± 9.28 | 189 | 48 | 22 | 13 |
| Normalized wall index | 0.55 ± 0.04 | 35 | 10 | 5 | 4 |
| Mean wall thickness (mm) | 0.75 ± 0.06 | 42 | 12 | 6 | 4 |
| Maximum wall thickness (mm) | 0.9 ± 0.07 | 39 | 11 | 6 | 4 |

Note: SD, standard deviation; ICA, internal carotid artery; MCA, middle cerebral artery; ACA, anterior cerebral artery; VA, vertebral artery; BA, basilar artery.
